# Plasma homocysteine and cerebral small vessel disease as possible mediators between kidney and cognitive functions in patients with diabetes mellitus

**DOI:** 10.1038/s41598-017-04515-w

**Published:** 2017-06-29

**Authors:** Mika Sonoda, Tetsuo Shoji, Yukinobu Kuwamura, Yujiro Okute, Toshihide Naganuma, Hideaki Shima, Koka Motoyama, Tomoaki Morioka, Katsuhito Mori, Shinya Fukumoto, Atsushi Shioi, Taro Shimono, Hisako Fujii, Daijiro Kabata, Ayumi Shintani, Masanori Emoto, Masaaki Inaba

**Affiliations:** 1 Department of Metabolism, Endocrinology and Molecular Medicine, Osaka City University Graduate School of Medicine, Osaka, Japan; 2 Department of Vascular Medicine, Osaka City University Graduate School of Medicine, Osaka, Japan; 3 Vascular Science Center for Translational Research, Osaka City University Graduate School of Medicine, Osaka, Japan; 4 Department of Urology, Osaka City University Graduate School of Medicine, Osaka, Japan; 5 Department of Internal Medicine III, Division of Nephrology, Osaka Medical College, Takatsuki, Japan; 6 Department of Premier Preventive Medicine, Osaka City University Graduate School of Medicine, Osaka, Japan; 7 Department of Diagnostic and Interventional Radiology, Osaka City University Graduate School of Medicine, Osaka, Japan; 8 Department of Drug and Food Evaluation, Osaka City University Graduate School of Medicine, Osaka, Japan; 9 Department of Medical Statistics, Osaka City University Graduate School of Medicine, Osaka, Japan

## Abstract

Cognitive impairment is more prevalent in those with decreased kidney function. We tested a hypothesis that an increased homocysteine and/or cerebral small vessel diseases (SVDs) mediate the link between kidney and cognitive functions in a cross-sectional study in 143 type 2 diabetes patients without diagnosis of dementia or prior stroke. The exposure and outcome variables were estimated glomerular filtration rate (eGFR) and cognitive performance evaluated with Modified Mini-Mental State (3 MS) examination, respectively. The candidate mediators were plasma homocysteine concentration, and SVDs including silent cerebral infarction, cerebral microbleed, periventricular hyperintensity, and deep and subcortical white matter hyperintensity by magnetic resonance imaging. In multiple regression models adjusted for 12 potential confounders, eGFR was positively associated with 3 MS score, inversely with homocysteine, but not significantly with the presence of any type of SVD. The association of eGFR with 3 MS remained significant when each of the SVDs was added to the model, whereas it disappeared when homocysteine was included in place of SVD. Mediation analysis indicated nearly significant mediation of homocysteine (P = 0.062) but no meaningful mediations of SVDs (P = 0.842–0.930). Thus, homocysteine, not SVDs, was shown to be the possible mediator between kidney and cognitive functions in patients with type 2 diabetes mellitus.

## Introduction

Cognitive impairment including dementia is seen at a high prevalence among patients with chronic kidney disease (CKD)^1, 2^. Although preventive measures of cognitive decline are eagerly desired, the precise mechanisms are not fully understood, by which cognitive performance is impaired in patients with decreased renal function.

In general, dementia is caused by neurodegenerative processes such as Alzheimer’s disease, or by vascular injury typically seen in post-stroke vascular dementia. The term vascular cognitive impairment (VCI) is used for all forms of cognitive deficits due to vascular disease from severe vascular dementia to mild cognitive impairment^3^. Recent studies^4^ have shown cerebral small vessel diseases (SVDs) as an independent predictor of incident dementia in the general population. Magnetic resonance imaging (MRI) is used to detect SVDs such as cerebral microbleed (CMB), silent cerebral infarction (SCI), periventricular hyperintensity (PVH), and deep and subcortical white matter hyperintensity (DSWMH). The etiologies of SVDs include the small arteries, arterioles, venules, and capillaries of the brain. SVDs are related to aging and hypertension, and some types of CMBs are related to amyloid angiopathy^5^. These vascular changes could contribute the development of VCI in subjects without history of clinically apparent stroke.

Impaired cognitive performance in patients with reduced kidney function may be explained by CKD-related VCI (CKD-VCI). CKD patients show impairment in various cognitive domains including executive function^6, 7^, the pattern being similar to vascular dementia rather than Alzheimer’s disease^8^. Patients with CKD, particularly those with end-stage renal disease (ESRD), are at an elevated risk for cardiovascular disease^9, 10^. Also, prior cardiovascular disease other than stroke was reported to be an independent factor of lower cognitive performance in hemodialysis patients without prior stroke^11^. As compared with healthy controls, patients with decreased renal function including ESRD are more likely to have SVDs such as CMB^12–15^, SCI^16^, PVH, and DSWMH^17, 18^. Since some studies have shown the association of the presence of SVDs with lower cognitive performance and cognitive impairment in CKD^17, 19, 20^, SVDs may be one of the links between kidney and cognitive function. To the contrary, a few studies showed that the association of reduced renal function with cognitive performance or dementia/cognitive impairment was independent of the presence of SVDs in the general population^4^ and in patients with diabetes mellitus^20^, suggesting additional pathways between the kidney and cognitive functions.

Hyperhomocysteinemia^21^ is one of the candidates explaining impaired cognitive function in patients with decreased kidney function^22^. Homocysteine is a sulfur-containing amino acid possessing toxicities against the vascular^23^ and also nervous cells^24^. Some studies showed the association of hyperhomocysteinemia with dementia^25^ and lowered cognitive performance^26^. So far, however, no study examined the possible contribution of homocysteine to impaired cognitive performance in patients with decreased kidney function.

**Figure 1:**
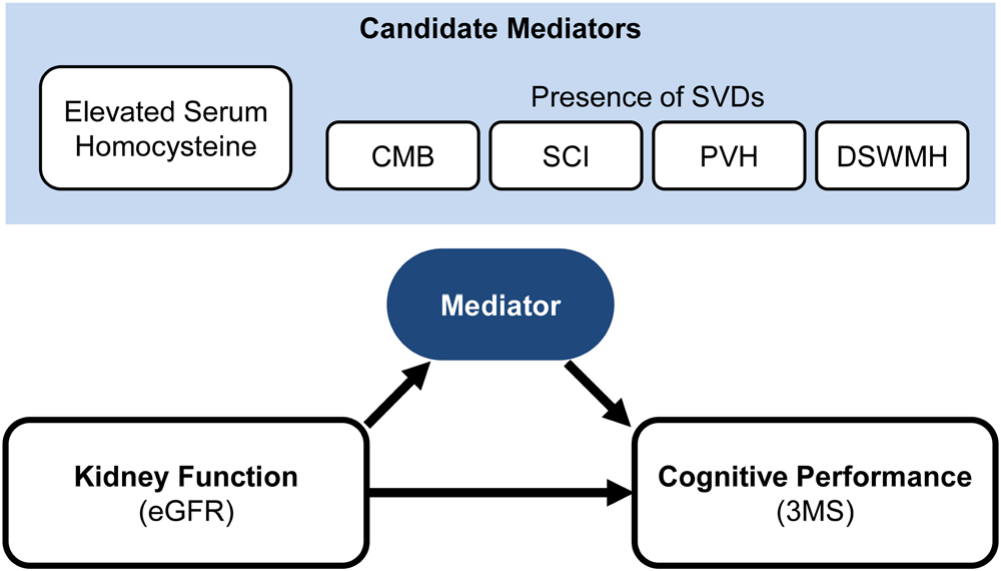
Study hypothesis. Decreased kidney function is associated with low cognitive performance. We hypothesized that elevated serum homocysteine and/or the presence of SVDs could mediate the link between kidney function and cognitive performance in patients with diabetes mellitus. The analysis was done by adjustment for potential confounders. Abbreviations are: eGFR, estimated glomerular filtration rate; CMB, cerebral microbleed; SCI, silent cerebral infarction; PVH, periventricular hyperintensity; DSWMH, deep and subcortical white matter hyperintensity; 3 MS, Modified Mini-Mental State examination.

The purpose of this study was to test a hypothesis that SVDs and/or homocysteine are the mediators in the link between the kidney and cognitive functions (Fig. 1).

## Results

### Selection and clinical characteristics of the participants

**Figure 2:**
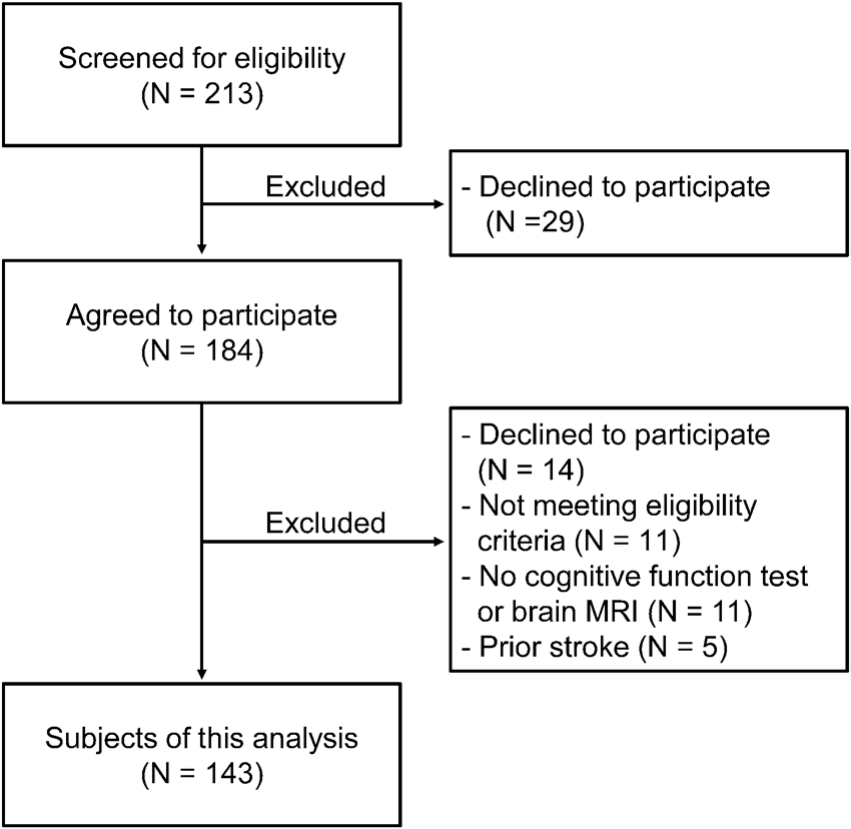
Selection of the participants. We excluded 5 participants because of the presence of prior stroke which was clinically symptomatic and diagnosed by imaging tests.

We recruited 143 participants from 213 patients screened for eligibility (Fig. 2). Table 1 summarizes the clinical characteristics of the participants. They all had type 2 diabetes mellitus, although the eligibility criteria did not exclude patients with type 1 diabetes. The median age was 71 years old, and 60.1% of the participants were men. The median of known duration of diabetes mellitus was 14 years. Their estimated glomerular filtration rate (eGFR; median 58.6; range 4.3 to 128.9 mL/min/1.73 m^2^) and urinary albumin to creatinine ratio (ACR; median 17; range 2 to 4451 mg/gCr) showed wide distributions. The median total score of Modified Mini Mental State (3 MS) Examination was 94 points. CMB, SCI, PVH and DSWMH were found in 23 patients (16.1%), 50 patients (35.0%), 106 patients (74.1%), and 130 patients (90.9%), respectively. The median plasma homocysteine level was 10.2 nmol/mL.

**Table 1:** Clinical characteristics of the study participants.

Measurement	Median (IQR), or Percentage
Age (years)	71 (64–75)
Sex (Male, %)	60.1
Duration of diabetes mellitus (years)	14 (8–20)
HbA1c (%)	8.0 (7.1–9.2)
eGFR (mL/min/1.73 m²)	58.6 (35.0–73.0)
Urine albumin to creatinine ratio (mg/gCr)	17 (6–215)
Total cholesterol (mg/dL)	178 (152–207)
Triglycerides (mg/dL)	118 (84–153)
High density lipoprotein cholesterol (mg/dL)	40 (35–53)
Non-high density lipoprotein cholesterol (mg/dL)	133 (108–158)
Serum albumin (g/dL)	4.1 (3.8–4.3)
Body mass index (kg/m^2^)	24.3 (21.9–26.9)
Systolic blood pressure (mmHg)	125 (110–139)
Diastolic blood pressure (mmHg)	65 (60–74)
Current smoker (%)	13.3
Education (≥college, %)	30.8
Use of medications	
Insulin injection (%)	50.0
Oral hypoglycemic agent (%)	73.4
Antihypertensive (%)	61.5
Statin (%)	48.3
Complications	
Hypertension (%)	67.1
Dyslipidemia (%)	86.7
Retinopathy (%)	28.2
Neuropathy (%)	50.4
Coronary artery disease (%)	11.2
Peripheral artery disease (%)	2.1
Aortic disease (%)	0.1
Congestive heart failure (%)	4.1
Any prior cardiovascular disease (%)	16.1
Apolipoprotein E_4_ positive (%)	21.7
Serum vitamin B_12_ (pg/mL)	493 (349–848)
Serum folate (ng/mL)	8.7 (6.0–11.2)
Plasma total homocysteine (nmol/mL)	10.2 (8.3–13.8)
Cognitive function	
3 MS total score (points)	94 (89–98)
Registration and recall (points)	20 (18–21)
Long–term memory (points)	5 (5–5)
Orientation (points)	20 (20–20)
Attention (points)	7 (5–7)
Verbal fluency and understanding (points)	21 (20–21)
Word retrieval (points)	10 (9–10)
Visuospatial skills (points)	10 (10–10)
Similarities (points)	4 (2–5)
TMT-B (seconds)	151 (108–244)
Cerebral SVDs	
CMB present (%)	16.1
SCI present (%)	35.0
PVH Grade 0, I, II, and III (%)	25.9/57.3/11.9/4.9
DSWMH Grade 0, 1, 2, and 3 (%)	9.1/28.7/48.2/14.0

The table gives percentage for categorical variables and median (IQR) for continuous variables. Abbreviations are: IQR, interquartile range; eGFR, estimated glomerular filtration rate; HbA1c, hemoglobin A1c; 3 MS, Modified Mini-Mental State examination; TMT-B, Trail Making Test B; SVD, small vessel disease; CMB, cerebral microbleed; SCI, silent cerebral infarction; PVH, periventricular hyperintensity; DSWMH, deep and subcortical white matter hyperintensity.

### Unadjusted relationship between the exposure, outcome and mediator variables

**Figure 3:**
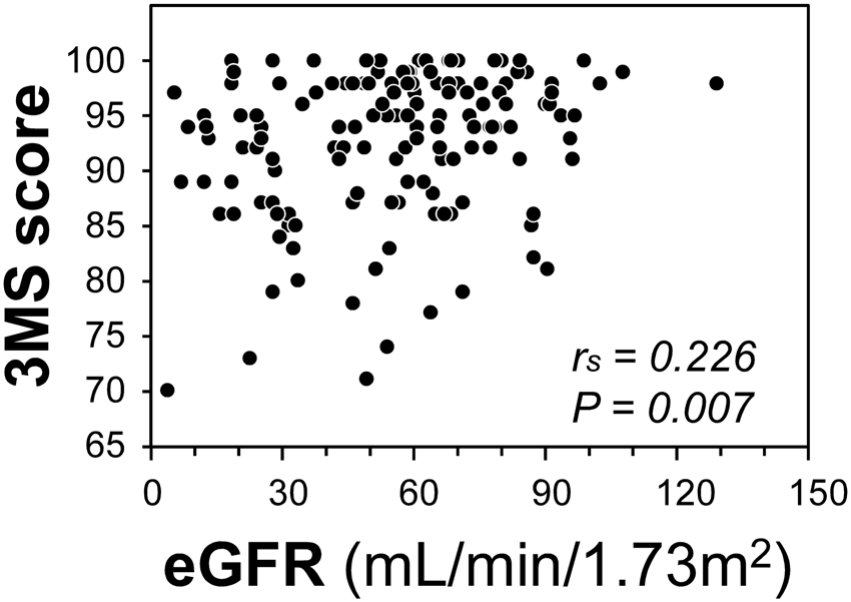
Correlation between eGFR and 3 MS total score in the total subjects. Abbreviations are: eGFR, estimated glomerular filtrationrate; 3 MS, Modified Mini-Mental State examination; r_s_, Spearman’s correlation coefficient; P, level of significance.

**Figure 4:**
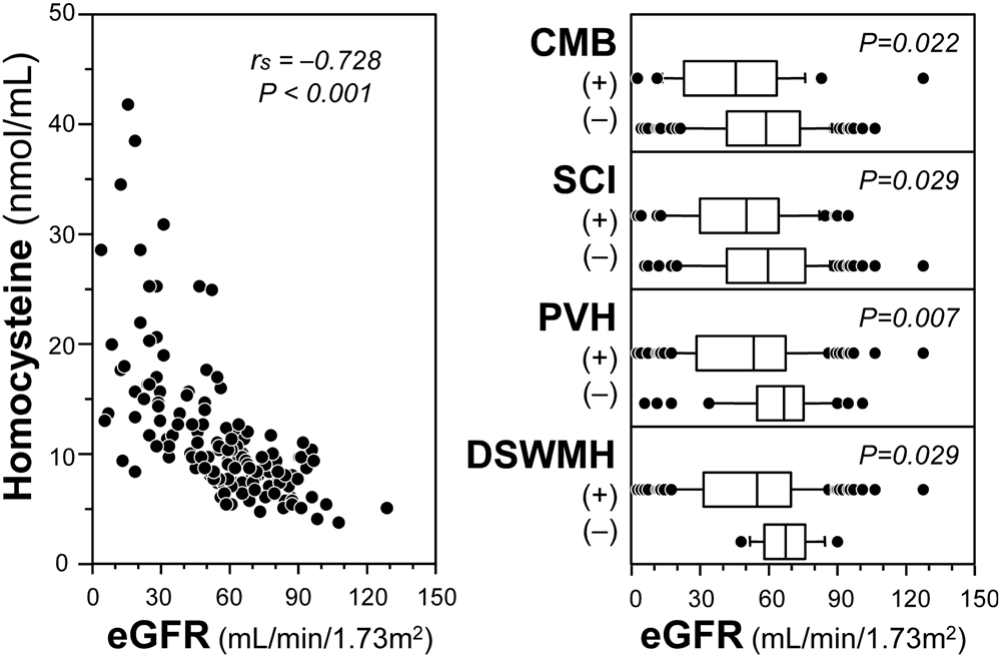
Unadjusted associations of eGFR with candidate mediators. Left panel shows the correlation between eGFR and serum homocysteine concentrations. Correlation was assessed by Spearman’s rank correlation test. Right panels compare eGFR levels between those with and without SVD. The box-and-whisker plots indicate 10th, 25th, 50th, 75th, and 90th percentile levels. Comparison between two groups was performed by Mann-Whitney U-test. Abbreviations are: 3 MS, modified mini-mental state; SVD, small vessel disease; CMB, cerebral microbleed; SCI, silent cerebral infarction; PVH, periventricular hyperintensity; DSWMH, deep and subcortical white matter hyperintensity; eGFR, estimated glomerular filtration rate; r_s_, Spearman’s rank correlation coefficient; P, level of significance.

**Figure 5:**
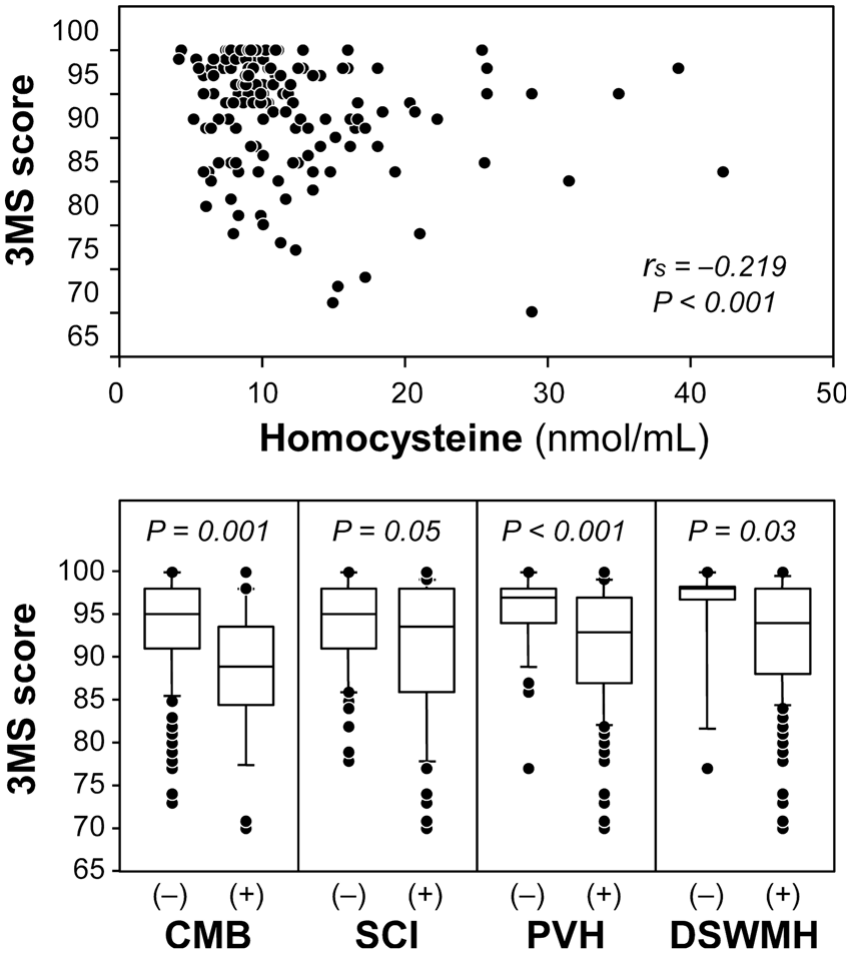
Unadjusted relationship of candidate mediators with 3 MS total score. Upper panel shows correlation between serum homocysteine concentration and 3 MS total score. Correlation was assessed by Spearman’s rank correlation test. Lower panels compare 3 MS total score between those with and without SVD. The box-and-whisker plots indicate 10th, 25th, 50th, 75th, and 90th percentile levels. Comparison between two groups was performed by Mann-Whitney U-test. Abbreviations are: SVD, small vessel disease; CMB, cerebral microbleed; SCI, silent cerebral infarction; PVH, periventricular hyperintensity; DSWMH, deep and subcortical white matter hyperintensity; eGFR, estimated glomerular filtration rate; r_s_, Spearman’s rank correlation coefficient; P, level of significance.

Total score of 3 MS was positively correlated with eGFR (Fig. 3). As shown in Fig. 4, eGFR showed significant associations with the candidate mediators. eGFR was inversely correlated with homocysteine level. eGFR was significantly lower in participants with CMB than those without CMB. This was also true for comparison between groups with and without SCI, PVH or DSWMH. Figure 5 shows the association of 3 MS with the candidate mediators. 3 MS total score was inversely correlated with homocysteine, and 3 MS total score was lower in patients with CMB than those without CMB. This was also true for comparison between groups with and without SCI, PVH or DSWMH.

Homocysteine concentrations were not significantly different between those with and without CMB [12.5 (6.7–18.3) vs. 10.2 (8.3–13.3) nmol/mL, P = 0.384], between those with and without SCI [10.5 (8.3–17.0) vs. 9.9 (8.3–13.2) nmol/mL, P = 0.166], between those with and without PVH [10.5 (8.3–15.1) vs. 9.7 (7.8–12.4) nmol/mL, P = 0.194], or between those with and without DSWMH [10.4 (9.3–14.7) vs. 9.2 (7.7–10.5) nmol/mL, P = 0.065].

### Mediation analysis

We explore the possibilities that SVDs and/or homocysteine are the mediator(s) in the link between eGFR and 3 MS total score using multiple regression models adjusted for 12 potential confounders (Table 2). First, the association between eGFR and 3 MS total score was significant in the adjusted model (Model 1). Regarding the association between eGFR and each of the five candidate mediators, only homocysteine showed a significant association with eGFR (Model 2). The associations of 3MS with CMB, SCI, PVH, and homocysteine remained significant after adjustment, whereas DSWMH did not (Model 3). The association of eGFR with 3 MS remained significant when the effect of the presence of CMB, SCI, PVH, or DSWMH was considered, whereas it was no longer significant when the effect of homocysteine was taken into account (Model 4). In the same model, the associations of 3 MS with CMB, SCI, PVH, and homocysteine were significant or nearly significant (P = 0.023–0.068), whereas the association of 3 MS with DSWMH was not. The mediation analysis indicated nearly significant mediation of homocysteine (P = 0.062) but no meaningful mediations of SVDs (P = 0.842–0.930).

**Table 2:** Mediation analysis.

Mediator	**Model 1:** eGFR → 3 MS		**Model 2:** eGFR → Mediator		**Model 3:** Mediator → 3 MS		**Model 4:** eGFR → Mediator → 3 MS				P for mediation analysis
	Coeff. of eGFR	P	OR or Coeff. of eGFR	P	Coeff. of Mediator	P	Coeff. of eGFR	P	Coeff. of Mediator	P	
CMB			0.774	0.498	−0.485	0.038	0.232	0.037	−0.449	0.052	0.926
SCI			1.227	0.455	−0.376	0.035	0.263	0.018	−0.401	0.023	0.930
PVH	0.248	0.027	0.715	0.264	−0.413	0.031	0.226	0.043	−0.374	0.049	0.928
DSWMH			0.735	0.593	−0.357	0.237	0.248	0.027	−0.357	0.230	0.842
Log Hcy			−0.275	<0.001	−0.309	0.006	0.089	0.534	−0.252	0.080	0.062

We tested a hypothesis that a higher homocysteine concentration and/or the presence of SVD (CMB, SCI, PVH or DSWMH) are mediator(s) of the association between eGFR and 3 MS in patients with diabetes mellitus. The multivariate models were adjusted for 12 potential confounders such as age, sex, apolipoprotein E4 isoform, prior cardiovascular disease, duration of diabetes mellitus, HbA1c, hypertension, dyslipidemia, log-transformed urine albumin to creatinine ratio, education, log-transformed serum vitamin B12, and log-transformed serum folate levels. **Model 1** indicates the association of eGFR with 3 MS without considering the effect of the mediator. **Mode 2** indicates the association of eGFR with the mediator by showing the adjusted odds ratios (per 1 standard deviation of eGFR) for CMB, SCI, PVH and DSWMH and the standardized regression coefficient for log-homocysteine. **Model 3** examined the association of the mediator with 3 MS. **Model 4** includes both eGFR and the mediator simultaneously to give the association of eGFR with 3 MS and the association of the mediator with 3 MS. P for mediation analysis indicates whether the coefficients of eGFR before (Model 1) and after inclusion of the mediator (Model 4) is significantly different. In all models, variable inflation factor was 3.462 or lower.

Abbreviations are: 3 MS, modified mini-mental state examination; eGFR, estimated glomerular filtration rate; CMB, cerebral microbleed; SCI, silent cerebral infarction; PVH, periventricular hyperintensity; DSWMH, deep and subcortical white matter hyperintensity; Hcy, homocysteine; OR, odds ratio; Coeff., standardized regression coefficient (ß); P, level of significance.

In these models, education level was consistently associated with 3 MS total score, whereas age was not an independent factor associated with 3 MS total score when the effects of eGFR, SVDs, and homocysteine were taken into consideration.

### Adjusted associations of various cognitive domains with eGFR, SVDs, and homocysteine

Using the same 12 covariates, we calculated the adjusted associations of eGFR, SVDs, and homocysteine with various domains of cognitive function (Table 3). Executive function was measured by Trail-Making Test B (TMT-B), whereas the other 8 cognitive domains were the subscales of the 3 MS total score. A lower score in “similarity” was associated with a lower eGFR and the presence of SVDs, but not with a homocysteine level, although the association with DSWMH did not reach statistical significance. A lower score in “orientation” was associated with the presence of CMB or SCI. A lower score in “word retrieval” was significantly associated with the presence of PVH, DSWMH and a higher homocysteine. The time to complete the task of TMT-B was associated with homocysteine level with marginal significance, but not significantly with eGFR or the presence of any form of SVDs.

**Table 3:** Adjusted associations of cognitive domains with eGFR, small vessel diseases, and homocysteine.

	eGFR	CMB	SCI	PVH	DSWMH	Log homocysteine
Registration and recall	0.085	−0.018	−0.096	0.005	−0.074	−0.061
Long-term memory	−0.038	0.031	−0.013	0.017	0.095	−0.064
Orientation	0.096	−**0**.**166** ^**#**^	−**0**.192*	−0.089	0.096	−**0**.**212** ^**#**^
Attention	0.116	−0.103	−0.056	−0.069	−0.086	−0.169
Verbal fluency and understanding	0.024	−0.086	0.019	−0.081	−0.059	−0.079
Word retrieval	0.126	−0.067	−0.014	−**0**.281*	−**0**.190*	−**0**.242*
Visuospatial skills	−0.095	−**0**.**164** ^**#**^	0.002	−0.079	−0.013	−0.046
Similarities	**0**.275*	−**0**.193*	−**0**.191*	−**0**.205*	−**0**.**145** ^**#**^	−0.165
TMT-B	−0.163	0.127	−0.027	−0.098	0.120	**0**.**191** ^**#**^

The table gives standardized regression coefficients (ß values) of eGFR, four types of SVD (SCI, CMB, PVH, or DSWMH), and log-transformed homocysteine levels for cognitive domains by multiple regression analysis adjusted for 12 covariates including age, sex, apoE4, prior cardiovascular disease, duration of diabetes mellitus, HbA1c, hypertension, dyslipidemia, log-transformed urine albumin to creatinine ratio, education, log-transformed serum vitamin B12, and log-transformed serum folate level. In this analysis, we coded SVDs as 0 (if absent) or 1 (if present). ^#^P = 0.06–0.09, *P < 0.05, **P < 0.01. Abbreviations are: eGFR, estimated glomerular filtration rate; 3 MS, Modified Mini-Mental State examination; TMT-B, Trail Making Test B; CMB, cerebral microbleed; SCI, silent cerebral infarction; PVH, periventricular hyperintensity; DSWMH, deep and subcortical white matter hyperintensity.

## Discussion

In the present study, we examined the possibilities that SVDs and/or homocysteine are the mediators of the link between renal function and cognitive performance in patients with diabetes mellitus. All of the five mediator variables were significantly associated with both eGFR and 3 MS in unadjusted analysis. However, in multiple regression analysis adjusted for 12 potential confounders, homocysteine showed a nearly significant mediation but none of SVDs showed meaningful mediation. Although not definitive, these results suggest that homocysteine, not SVDs, is a mediator between kidney and cognitive functions in patients with type 2 diabetes mellitus.

In search of the evidence that vascular abnormalities are involved in the cognitive deficit in decreased kidney function or in CKD, some previous studies explored the possible association of cognition with past history of atherosclerotic cardiovascular disease such as stroke and/or transient ischemic attack^27^ and coronary heart disease^28^. However, the history of these cardiovascular diseases did not explain the reduced cognitive function in patients with decreased kidney function^27^ or in predialysis patients with CKD^28^. In line with these reports, we found no significant association between 3 MS score and prior cardiovascular disease other than stroke in patients with diabetes mellitus without prior stroke. These studies raise a possibility that cognitive problems in patients with reduced kidney function are not directory related to atherosclerotic cardiovascular diseases, but rather more closely to non-atherosclerotic vascular changes including microvascular alterations, both of them being prevalent in patients with decreased kidney function^10^.

More recent studies have examined the association between cognition and SVDs using MRI. Yamamoto *et al*.^19^ reported that the presence and degree of SVDs and a lower eGFR were independently associated with cognitive impairment in patients with symptomatic lacunar infarction. Umemura *et al*.^20^ reported that the association of renal function with cognitive performance was independent of SVDs (SCI or WMHs) in 79 patients with diabetes mellitus. In addition to these cross-sectional studies, Miwa *et al*.^29^ performed a cohort study in the general Japanese population with vascular risk factors, showing that the presence of CKD and the presence of SVDs (SCI or WMHs) at baseline were independently predictive of incident dementia. They^4^ also reported that the presence of multiple or mixed CMBs at baseline predicted all-cause dementia. Our study has shown that cognitive performance was independently associated with eGFR and the presence of SVDs, namely SCI, CMB, PVH, but not DSWMH, in patients with diabetes mellitus. Thus, the present study provides further support for the involvement of SVDs in lower cognitive performance. Importantly, however, these studies unanimously showed that the presence of SVDs did not fully explain the association between eGFR and cognitive function.

Then, we aimed to examine other factors than SVDs that could explain the link between eGFR and cognitive function. Bugnicourt *et al*.^22^ proposed a list of the possible contributors to the cognitive decline in CKD, such as inflammation, hypercoagulable state, oxidative stress and hyperhomocysteinemia. Among these candidates, we focused on homocysteine in this study. In addition to its toxic effects on the vascular system^23, 30, 31^, homocysteine was shown to have a direct neurotoxic effect at an elevated level through its agonistic action at the glutamate binding site of the N-methyl-D-aspartate receptor^32^. Also, homocysteine could cause brain damage by many other mechanisms such as disturbed protein methylation, promotion of calcium influx, and tau protein accumulation, finally resulting in apoptosis and neuronal death^24^. Furthermore, there is a study reporting an association between plasma homocysteine and amyloid beta protein levels in some conditions^33^. Some^25, 26^ but not all studies^34, 35^ found significant association of plasma homocysteine with cognitive deficit and dementia in elderly people. So far, however, no study has examined the role of homocysteine in decreased cognitive performance in association with decreased kidney function. In our study adjusted for 12 potential confounders, 1) eGFR was positively associated with 3 MS (P = 0.027), 2) eGFR was inversely associated with homocysteine (P < 0.001), 3) homocysteine was inversely associated with 3 MS (P = 0.006), and 4) the association of eGFR and 3 MS was no longer significant when homocysteine was simultaneously included in the model (P = 0.534). These results of homocysteine fulfill the criteria of a mediator variable by Baron and Kenny^36^, although the mediation of homocysteine did not reach statistical significance (P = 0.062). Thus, we could not definitively conclude that homocysteine is the mediator in the link between renal function and cognitive performance in patients with diabetes mellitus. However, we interpret these data to indicate that homocysteine is a possible mediator.

We noticed that eGFR, homocysteine and the presence of SVDs show different pattern of association with cognitive domains. Most importantly, executive function measured by TMT-B was preferentially associated with eGFR and homocysteine. This confirms the previous reports^6, 7^ that the association between reduced renal function and impaired executive function. Since the lower performance in executive function in CKD is similar to the pattern seen in vascular dementia^8^, the impaired executive function has been considered as an evidence for vascular mechanisms in cognitive decline in CKD. However, we found no association between TMT-B and any type of SVDs. A very recent report^37^ has revealed the close association between homocysteine and the score of Digital Symbol Substitution test, a measure of executive function. Thus, the impaired executive function in subjects with decreased eGFR may be explained not by SVDs but by an increased homocysteine level.

Nutritional factors may affect cognitive function. In the participants of this study, 3 MS total score did not show significant correlation with either BMI (r_s_ = −0.024, P = 0.769) or serum albumin (r_s_ = 0.114, P = 0.175). Deficiencies of vitamin B_6_, B_12_, and/or folate are known to affect serum homocysteine level and cognitive function^38^. Because we did not use light-resistant containers for serum sampling and storage, we could not surely assess vitamin B_6_ deficiency. We identified two participants having serum vitamin B_12_ level below 200 pg/mL and two participants with serum folate level below 3 ng/mL. We included these vitamin levels in univariate and multivariate analyses, but they did not show meaningful associations with 3 MS.

This study has several limitations. First, because of a cross-sectional design, the associations do not necessarily indicate causality. Second, we did not use an automated image analyzer for the evaluation of SVDs, but a traditional method by classifying the abnormalities on brain MRI. Nonetheless, we were able to detect the significant association between SVDs and cognitive performance. Thus, the results may be more easily applied to daily clinical practice in which sophisticated software for image analysis is not always available. Third, we used the eight subscales of 3 MS for the assessment of various cognitive domains, not an extensive neurocognitive battery. Fourth, the relatively small sample size of this study may have prevented from more definite results of the mediation analysis. Fifth, we did not measure hippocampal volume. And finally, since all the subjects of this study had diabetes mellitus, it is unclear whether the present results could be generalized to non-diabetic subjects.

In conclusion, we confirmed the significant associations of cognitive function with the presence SVDs and plasma homocysteine level in patients with diabetes mellitus. We did not find the mediations of SVDs in the link between kidney function and cognitive performance, but plasma homocysteine appeared to be a possible mediator. These results suggest both vascular and non-vascular mechanisms for lowered cognitive performance in individuals with decreased kidney function. Since SVDs and hyperhomocysteinemia are prevalent in subjects with decreased renal function, and since these factors were more important determinants of cognitive performance than chronological age as found in this study, we may be able to prevent cognitive decline by targeting these factors. Clearly, further studies are needed to confirm the current observations with a larger sample size and by other settings.

## Methods

### Study design and participants

This is a cross-sectional study. The inclusion criteria were (1) Patients with diabetes mellitus but not yet receiving renal transplantation or dialysis treatment, (2) Out-patients or In-patients of Osaka City University Hospital, (3) Patients of 50 years old or older, and (4) Patients who gave written informed consent. The exclusion criteria were (1) Patients hospitalized due to acute or severe illness, (2) Patients who are receiving medication for dementia, (3) Those who are judged inappropriate by an attending physician such as patients who had been diagnosed to have dementia. We planned to recruit participants from August 2012 to March 2016. For this analysis, we excluded 5 participants because of the presence of prior stroke which was clinically symptomatic and diagnosed by imaging tests.

### Ethical consideration

This study protocol was in accordance with Declaration of Helsinki, approved by the Ethics Committee, Osaka City University Graduate School of Medicine (No. 2428), and registered at University hospital Medical Information Network (UMIN) Clinical Trial Registry (Identifier UMIN000009233). All participants gave written informed consent prior to the study.

### Measurement of cognitive performance

We used 3 MS and Trail Making Test Part B (TMT-B) for the assessment of global cognitive function and executive function, respectively. These tests were performed by a single examiner (M.S.) within one month prior to MRI studies.

3 MS examination was developed by Teng *et al*.^39^. As compared with the original Mini-Mental State Examination (MMSE), 3 MS has improved features such as the extended ceiling and floor of the test, the wider range of cognitive ability, and the reliability and validity of the scores^39^. It consists of 15 questions, giving scores for 8 cognitive domains including “registration and recall” (immediate and delayed; full mark 21), “long-term memory” (date and place of birth; full mark 5), “orientation” (temporal and spatial; full mark 20), “attention” (mental reversal; full mark 7), “verbal fluency and understanding” (naming, repetition and writing a sentence, reading and obeying an order, three-stage command; full mark 21), “word retrieval” (four-legged animals; full mark 10), “visuospatial skills” (copying two pentagons; full mark 10), and “similarities” (a point in common between two words; full mark 6). We calculated the total scores (full mark 100), as well as the scores for each domain. The lower 3 MS score is considered worse in cognitive performance. We used a Japanese version of 3 MS.

TMT-B^40^ consists of circled numbers (1 to 13) and circled letters (A to L) distributed on a sheet. The patients were asked to draw lines connecting the numbers and letters alternately in an ascending pattern (namely, 1-A-2-B-3-C, and so on) as fast as they can without lifting the pencil from the sheet. The time to finish the task was measured in second using a stop watch. The higher TMT-B score is considered worse in executive function. In this study, we used a sheet on which circled numbers and circled Japanese Hiragana letters, instead of circled alphabets.

### Definitions of SVDs on MRI

All MRI examinations were performed using a 1.5-Tesla MRI unit (Avanto; Siemens, Erlangen, Germany) with a 12-channel head coil. All sequence protocols were for 2D axial images. MR imaging protocol included the following sequences in all patients: conventional spin-echo (SE) T1-weighted imaging (Repetition Time (TR) = 591 ms, Echo time (TE) = 11 ms, matrix = 256 × 230, NEX = 1), fast spin-echo (FSE) T2-weighted imaging (TR = 4000 ms, TE = 90 ms, matrix = 448 × 314, NEX = 1), fluid-attenuated inversion recovery (FLAIR) imaging (TR = 8000 ms, TE = 105 ms, inversion time (TI) = 2500 ms, matrix = 256 × 208, NEX = 1), gradient echo T2*-weighted imaging (TR = 650 ms, TE = 19 ms, matrix = 256 × 204, NEX = 1, flip angle = 20 degrees), and single-shot echo-planar diffusion-weighted imaging (DWI) (TR = 3000 ms, TE = 75 ms, matrix, 128 × 96, NEX = 6) with a b value of 0 and 1000 s/mm^2^. Other sequence parameters of all MR imaging were as follows: FOV = 22 × 22 cm^2^; section thickness, 5 mm with 1 mm gap, 25 sections.

Since we excluded patients with prior stroke, and no one had history of transient ischemic attack, all cerebral lesions detected by MRI were considered as silent ones. CMBs were defined as small signal loss areas on T2*-weighted MRI. We ruled out symmetric signal losses because of possibility of being calcification or flow void artifact of the pial blood vessels. SCIs were defined as focal lesions, 3 mm or larger in diameter, which were prominently hyperintense on T2-weighted images, hypointense on T1-weighted images, and hypointense and surrounded by a hyperintense rim on FLAIR images. WMHs, or lesions called leukoaraiosis, were defined as abnormalities which were hyperintense on T2-weighted images and prominently hyperintense on FLAIR images. By their locations, WMH was classified into periventricular WMH (PVH) and deep subcortical WMH (DSWMH). According to Fazekas *et al*.^41^, PVH was graded as 0 (absence), I (“caps” or “pencil-thin lining”), II (smooth “halo”), or III (irregular PVH extending into the deep white matter). DSWMH was graded as 0 (absence), 1 (punctuate foci), 2 (beginning confluence of foci), or 3 (large confluent areas).

We examined the inter-rater agreement between a nephrologist (M.S.) and a board-certified neuroradiologist (Ta.S.) in a preliminary study with MR images from 20 participants, and found complete agreement (kappa = 1.00) for the presence or absence of CMB, SCI, PVH, or DSWMH. Then, two nephrologists (M.S. and Y.K.) independently rated the images from the total 143 participants, with kappa coefficients of 0.85 for CMB, 0.67 for SCI, 0.80 for PVH, and 0.56 for DSWMH. For cases of discordance, they discussed until reaching consensus.

### Kidney function, albuminuria, and homocysteine

All blood and urine samples were collected in the morning after an overnight fast for 12 hours. Serum and urinary creatinine levels were measured by an enzymatic method. Urinary albumin was measured by an immunoturbidimetry. Renal function was assessed by eGFR using a formula for the Japanese^42^. Urinary albumin to creatinine ratio was calculated as an index of albuminuria. Plasma total homocysteine was measured by high performance liquid chromatography^43^ at SRL, Inc., Tokyo. The reference range of homocysteine was 3.7 to 13.5 nmol/mL.

### Apolipoprotein E isoform typing

We determined serum apo E isoforms by isoelectric focusing followed by immunoblotting with anti-human apo E antibody (SRL Inc, Tokyo). For statistical analysis, the isoforms were coded as either apoE4 positive (high risk for Alzheimer’s disease^44^) or apoE4 negative.

### Other information

We collected information on age, sex, height, weight, duration of diabetes, current medications, past history of cardiovascular disease (coronary artery disease, peripheral artery disease, aortic disease, and congestive heart failure requiring hospitalization), education level (graduate school, college, high school, junior high school or lower), smoking habit, and laboratory data by asking the participants and/or by reviewing their medical records. Hypertension was defined by systolic blood pressure ≥140 mmHg, diastolic blood pressure ≥90 mmHg, or use of medication for hypertension. Dyslipidemia was defined by low density lipoprotein cholesterol ≥140 mg/dL, high density lipoprotein cholesterol <40 mg/dL, triglyceride ≥150 mg/dL, non-high density lipoprotein cholesterol ≥170 mg/dL^45^, or use of a statin. Serum levels of vitamin B12 and folate were measured by chemiluminescence enzyme immunoassays (SRL Inc, Tokyo).

### Statistics

We summarized continuous variables as medians (interquartile ranges, IQRs) and categorical variables as numbers and percentages. Comparison of continuous variables between groups was performed by Mann-Whitney U-test. Correlation was examined by Spearman’s rank correlation test.

The exposure and outcome variables were eGFR and 3 MS total scores, respectively, and the candidate mediators were plasma homocysteine, the presence of each SVD (CMB, SCI, PVH, and DSWMH). First, we examined unadjusted associations between the exposure, mediator, and outcome variables. Then, the adjusted associations of these variables were examined using multiple regression model and logistic regression model which included 12 potential confounders (age, sex, apoE4, prior cardiovascular disease, known duration of diabetes mellitus, HbA1c, hypertension, dyslipidemia, urine albumin to creatinine ratio, education level, serum vitamin B_12_, and serum folate level). Urine albumin to creatinine ratio, plasma homocysteine, serum vitamin B_12_ and folate levels were log-transformed to fit the models. Mediation was assessed by the criteria by Baron and Kenny^36^ and by the method by Imai, Keele, and Tingley^46^. In model 1 (eGFR → 3 MS), the association between eGFR and 3 MS total score was examined by multiple regression analysis, and the standardized regression coefficient (ß) of eGFR was given. In model 2 (eGFR → mediator), the association between eGFR and the mediator variable was assessed by multiple linear regression analysis and logistic regression analysis, and ß-coefficient and odds ratios (per 1 standard deviation of eGFR) were calculated, respectively. In model 3 (mediator → 3 MS), the association between the mediator and 3 MS was evaluated by multiple linear regression analysis. In model 4 (eGFR → mediator → 3 MS), the associations of eGFR and the mediator with 3 MS were simultaneously evaluated. Finally, in the mediation analysis, we compared the ß-coefficients of eGFR for 3 MS before and after inclusion of the mediator variable.

As additional analysis, we calculated ß-coefficients of eGFR and the candidate mediators for cognitive domains (8 subscales of 3 MS and TMT-B) adjusted for the 12 covariate as mentioned above.

A two-sided P-value < 0.05 was considered statistically significant. These statistical calculations were performed by JMP software (version 11, SAS Institute Japan, Tokyo), and mediation analyses were conducted with R software (version 3.3.2, R Foundation for Statistical Computing, Vienna, Austria) using the “mediation” package on Windows personal computers.
